# Subarachnoid Haemorrhage in a Patient With Suspected Infective Endocarditis in a District General Hospital: A Case Report-Based Literature Review

**DOI:** 10.7759/cureus.21602

**Published:** 2022-01-25

**Authors:** Zahid Khan, Vinod Warrier, SyedAun Muhammad, Charlie Mckechnie

**Affiliations:** 1 Cardiology, Royal Free Hospital, London, GBR; 2 Internal Medicine, Mid and South Essex NHS Foundation Trust, Southend on Sea, GBR; 3 Cardiology, Mid and South Essex NHS Foundation Trust, Southend on Sea, GBR

**Keywords:** spontaneous intracerebral haemorrhage, echocardiography, valvular heart disease, heart failure, left main aneurysm, mycotic disease, prolonged antibiotics, streptococcus bovis, staphylococcus aureus bacteremia, infective endocarditis

## Abstract

We describe the case of a 70-year-old lady who presented to a district general hospital during an evening with fevers, feeling generally unwell. She was found to have weakness in her left upper limb and went on to have tonic-clonic seizures whilst in the Accident and Emergency Department. CT scan of the brain showed subarachnoid haemorrhage, in absence of headache, in the right frontal, superior parietal and left occipital regions. Her C-reactive protein level was elevated at 426 mg/L and her urine dip was normal. Chest radiograph showed small bilateral pleural effusions. In addition to the above-mentioned findings on clinical examination, she also had pansystolic murmur although did not have any other feature of infective endocarditis (IE). In view of the above findings, normal chest examination and no urinary symptoms, the decision was made to treat this as a case of IE empirically. She subsequently went into fast atrial fibrillation requiring direct current (DC) cardioversion and intensive care unit admission due to hypotension. The next day, echocardiography confirmed vegetations and blood cultures were positive for *Staphylococcus aureus*. Her MRI scan of the brain confirmed parenchymal haemorrhages and haemorrhagic infarcts. She completed a 6-week course of antibiotics and clinically improved. Despite being critically unwell, appropriate antibiotics were initiated within hours of her admission in view of clinical suspicion of underlying IE, which aided her recovery.

## Introduction

Infective endocarditis (IE) most commonly presents with low-grade fever, night sweats, general malaise, however, patients may also present with encephalopathy, headache, seizures, stroke and meningitis [1]. Stroke in patients with IE may be haemorrhagic or ischaemic and is commonly secondary to cardio-embolism, septic emboli or mycotic aneurysms. The three monthly mortality of patients with IE significantly increases in patients with neurological features (24% vs 10%) compared to patients without neurological complications [2]. Intracranial haemorrhage (ICH) is mostly caused by mycotic aneurysms, accounting for 5% of neurologic complications associated with IE and rupture of mycotic aneurysms is associated with 80% mortality [1,2].

Prompt diagnosis of IE and early initiation of appropriate antibiotics, to improve patients’ chances of survival is, therefore, vital in patients with suspected IE [3]. Nevertheless, it can be very challenging for acute medicine physicians to promptly diagnose IE without the availability of certain out of hours facilities such as echocardiogram. This also highlights the fact that echocardiography is an essential skill for acute medicine physicians. It is also important to highlight that thorough clinical assessment is essential for the early management of patients with suspected IE who may present with neurological complications. In addition, the use of diagnostic criteria such as “Major Duke Criteria” can be useful in absence of essential imaging such as echocardiography findings [4]. This will facilitate early treatment of patients with suspected IE and help in better management of these patients, leading to a good outcome. We present this case report of a 70-year-old woman for educational purposes who presented to a district general hospital (DGH) with a subarachnoid haemorrhage secondary to IE.

## Case presentation

A 70-year-old woman was brought to the emergency department (ED) of a DGH during a late evening for being generally unwell. She was diagnosed with a urinary tract infection one week prior, for which she was treated with oral antibiotics. Her condition worsened in the preceding four days and she started to develop high fever, weakness and hypoesthesia in the left upper limb and deterioration in her vision. On arrival at the hospital, she experienced a self-terminating generalised tonic-clonic seizure. She developed atrial fibrillation with fast ventricular response in ED and was direct current (DC) cardioverted initially in view of systemic instability as the patient was very hypotensive. Following this, she developed two episodes of supraventricular tachycardia (SVT) and she received intravenous (IV) adenosine following which she reverted back to normal sinus rhythm (NSR) both times.

Past medical history was limited to osteopenia, rotator cuff shoulder syndrome and unclear valvular regurgitation reported by the patient, identified during an obstetric check-up 46 years previously. Examination in ED revealed a pansystolic murmur with mid systolic click and maximum intensity heard at the apex, capillary refill time (CRT) of 3 seconds and tiny 3-4 mm non-tender, non-erythematous lesions on both feet, suspicious for Janeway lesions and her Glasgow Coma Scale (GCS) score was 14/15 (E4 M6 V4). She was not able to move her left arm against gravity and exhibited reduced sensation. An electrocardiogram (ECG) showed sinus tachycardia with ventricular ectopics. Bilateral small pleural effusions were visible on her chest radiograph. Blood tests revealed raised inflammatory markers as shown in Table 1. Her vasculitis screen was normal and erythrocyte sedimentation rate (ESR) was 27 mm/h (Normal range for women 0-29 mm/h).

**Table 1:** Blood tests for patient on day 1

Blood Test	Normal Value	Day 1
White cell count	20	3.8-11 × 10^9^/L
Neutrophil	17	2-7.5 × 10^9^/L
Haemoglobin	122	115-155 g/L
Urea	7.8	2.5-7.8 mmol/L
Creatinine	104	45-84 µmol/L
Sodium	136	133-146 mmol/L
Potassium	4.5	3.5 -5.3 mmol/L
Platelet	70	150-400 × 10^9^/L
C-reactive protein	426	0-5 mg/L
Alkaline phosphatase	209	30-130 IU/L
Alanine transaminase	91	0-33 IU/L

**Figure 1:**
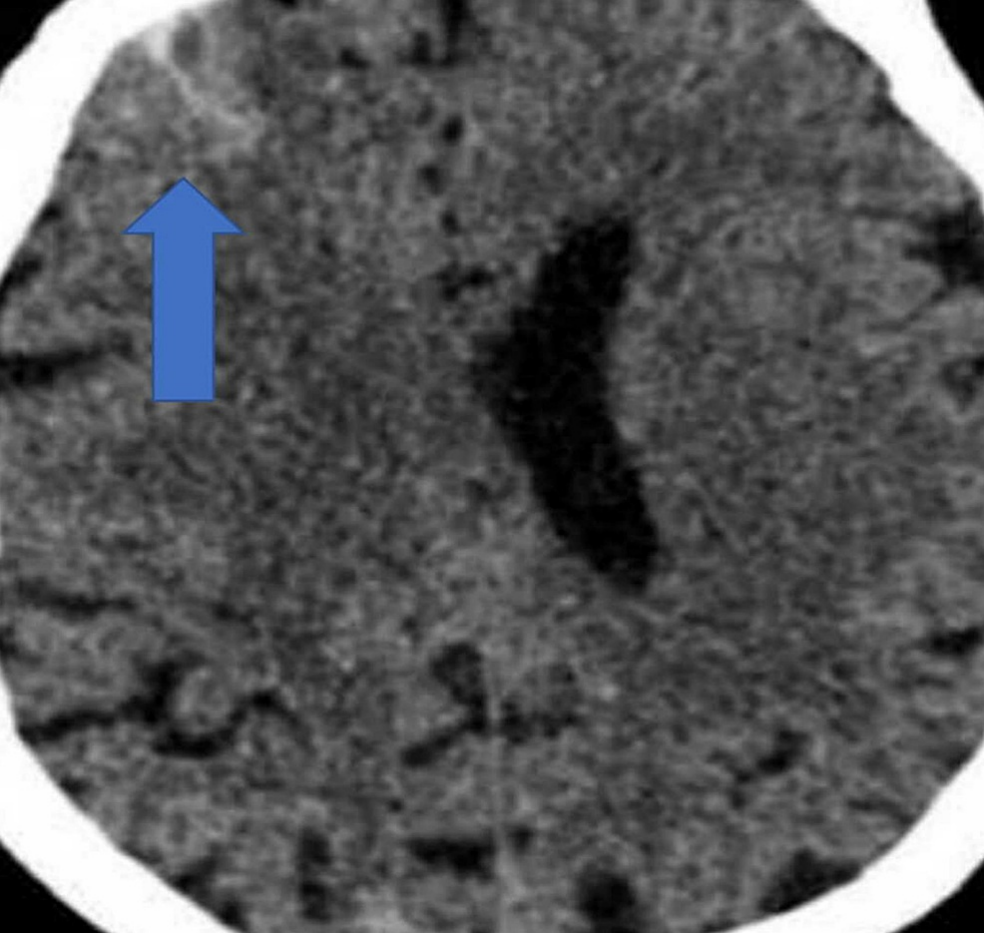
Non-contrast CT head axial view showing subarachnoid haemorrhage (arrow) in the right frontal, superior parietal regions

**Figure 2:**
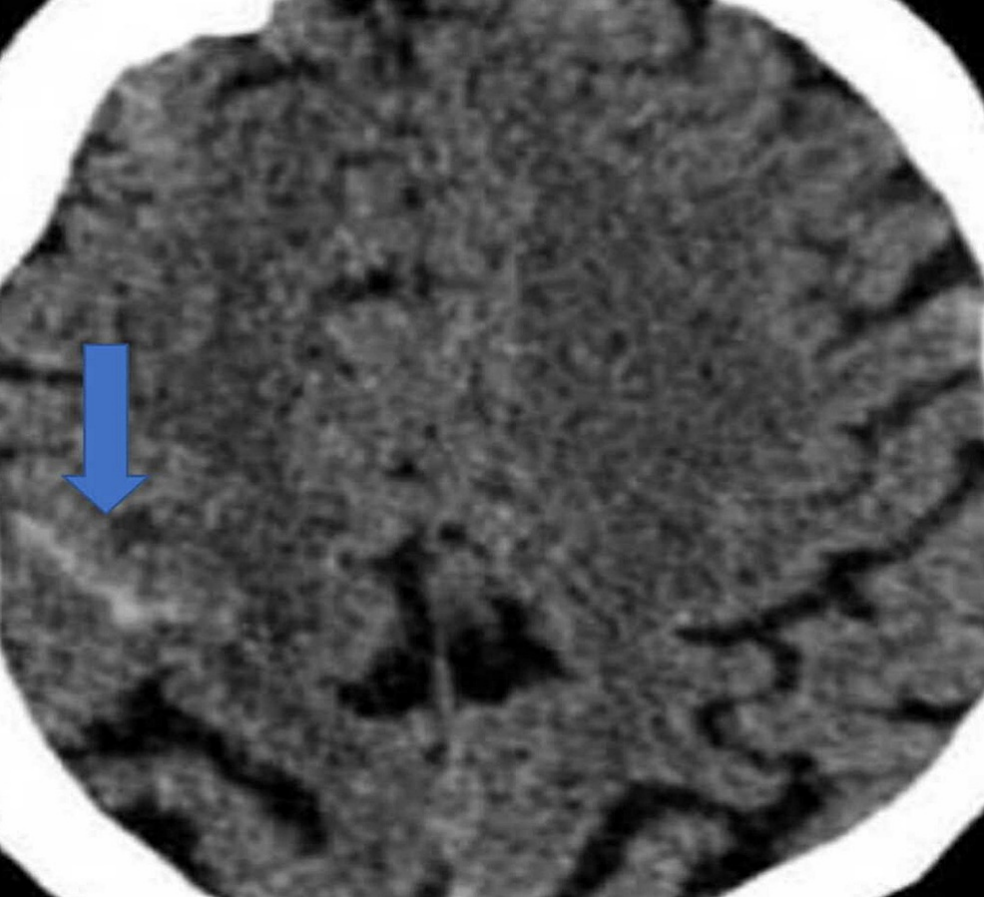
Non-contrast CT head axial showing subarachnoid haemorrhage (arrow) in the right frontal, superior parietal regions

An urgent CT head was arranged, which showed an acute subarachnoid haemorrhage (SAH) in the right frontal, superior parietal regions (Figures 1-2).

**Figure 3:**
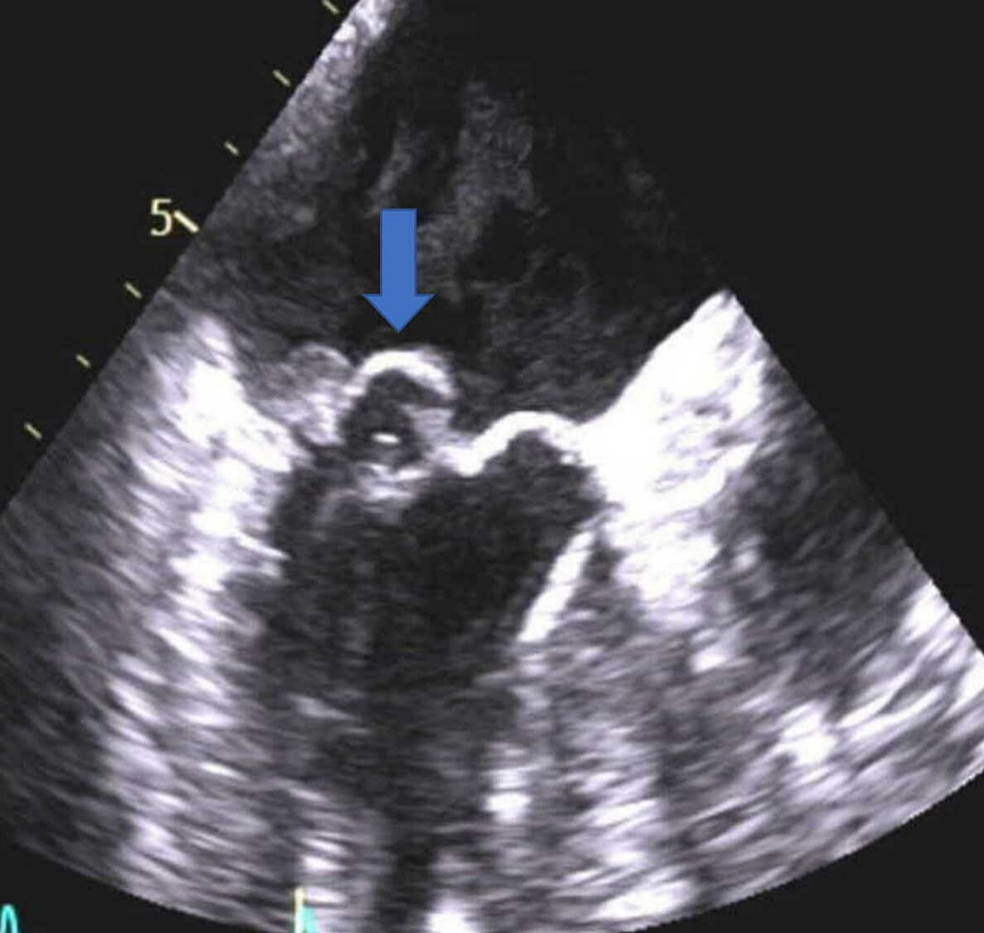
Transoesophageal echocardiogram showing mitral valve thickening and vegetation

**Figure 4:**
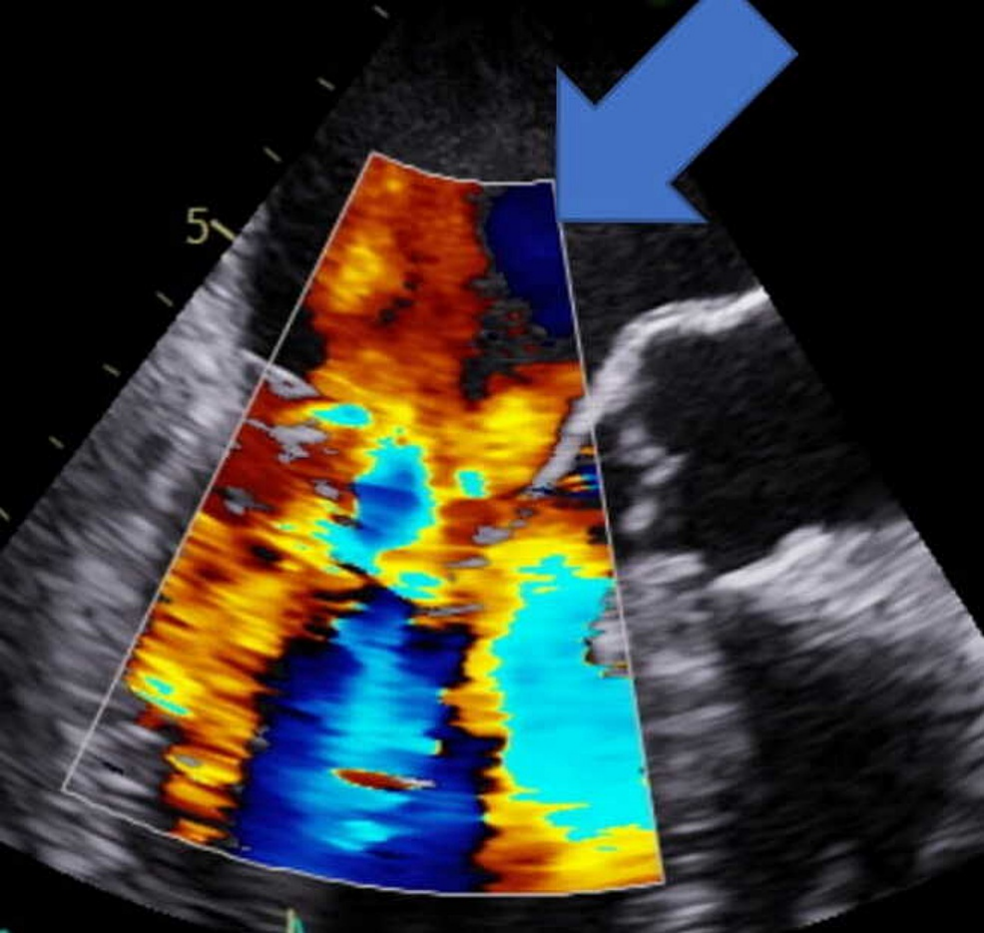
Transoesophageal echocardiogram showing severe mitral valve regurgitation

She had persistently elevated inflammatory markers despite one-week treatment with antibiotics pre-admission for urinary tract infection (UTI) along with continuous fever spikes, and night sweats for the last four nights. There was no obvious source of infection identified in this patient and in view of her thrombocytopenia along with pansystolic murmur and raised inflammatory markers, accompanied by SAH in the absence of other sources of infection, an empirical diagnosis of IE was proposed with cerebral mycotic aneurysms. The likelihood of UTI was low as this patient had no nitrites in her urine, however, had haematuria on the dip and urine midstream specimen (MSU). She had two small bilateral pleural effusions on chest radiograph (CXR), however, they were not believed to be the source of infection. In view of the above findings, a clinical decision was made to cover this patient empirically with IV vancomycin for IE till she gets a departmental echocardiogram to rule it out. Bedside transthoracic echocardiogram followed by transoesophageal echocardiogram (TOE) the next morning confirmed infective vegetation and thickening of both mitral valve leaflets, with regurgitation velocity of 1.2 m/s (Figures 3-4).

**Figure 5:**
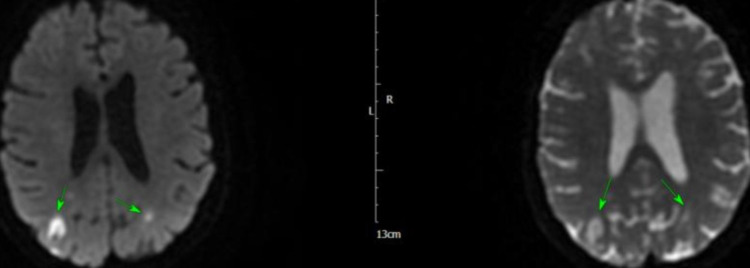
MRI of the head showing subarachnoid haemorrhage (arrows)

**Figure 6:**
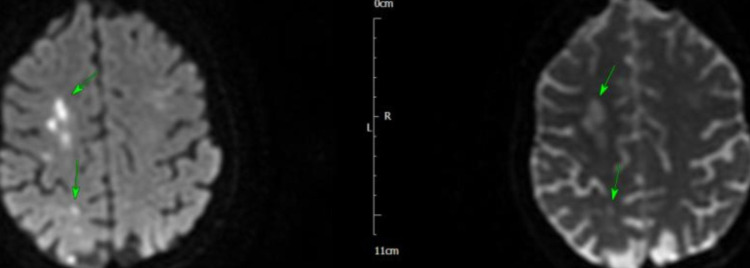
MRI of the head showing haemorrhagic infarcts (arrows)

**Figure 7:**
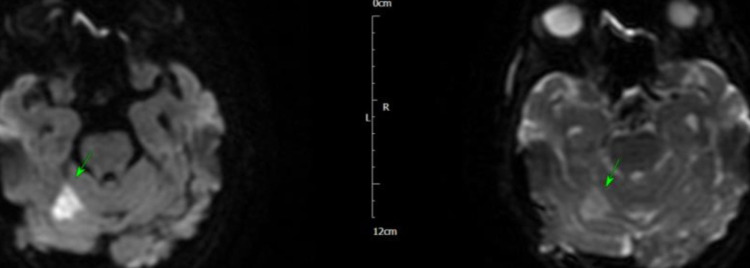
MRI of the head showing subarachnoid haemorrhage (arrows)

**Figure 8:**
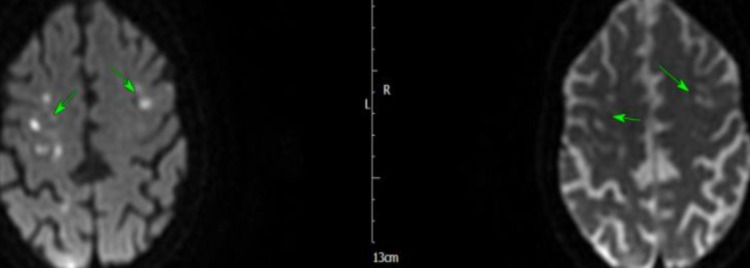
MRI of the head showing subarachnoid haemorrhage (arrows)

The next morning, she became tachycardic and hypotensive, and ECG revealed atrial fibrillation (AF) with a fast ventricular response, that responded to amiodarone infusion. She became more unwell later in the day and was transferred to intensive therapy unit (ITU) for inotropic support to maintain blood pressure and reasonable urine output. She had full septic screen on admission and blood cultures from admission grew *Staphylococcus aureus* (SA) sensitive to flucloxacillin. She was discussed with the microbiologist on call and antibiotic management was changed to IV flucloxacillin. Over the next few days, the patient showed improvement and her inflammatory markers started to improve, and she was stepped down from ITU to coronary care unit (CCU). She subsequently had MRI of the head, that showed multiple areas of peripheral parenchymal haemorrhage, with no sign of aneurysm or arteriovenous malformation, and the findings were rather in keeping with septic emboli secondary to IE (Figures 5-8).

**Figure 9:**
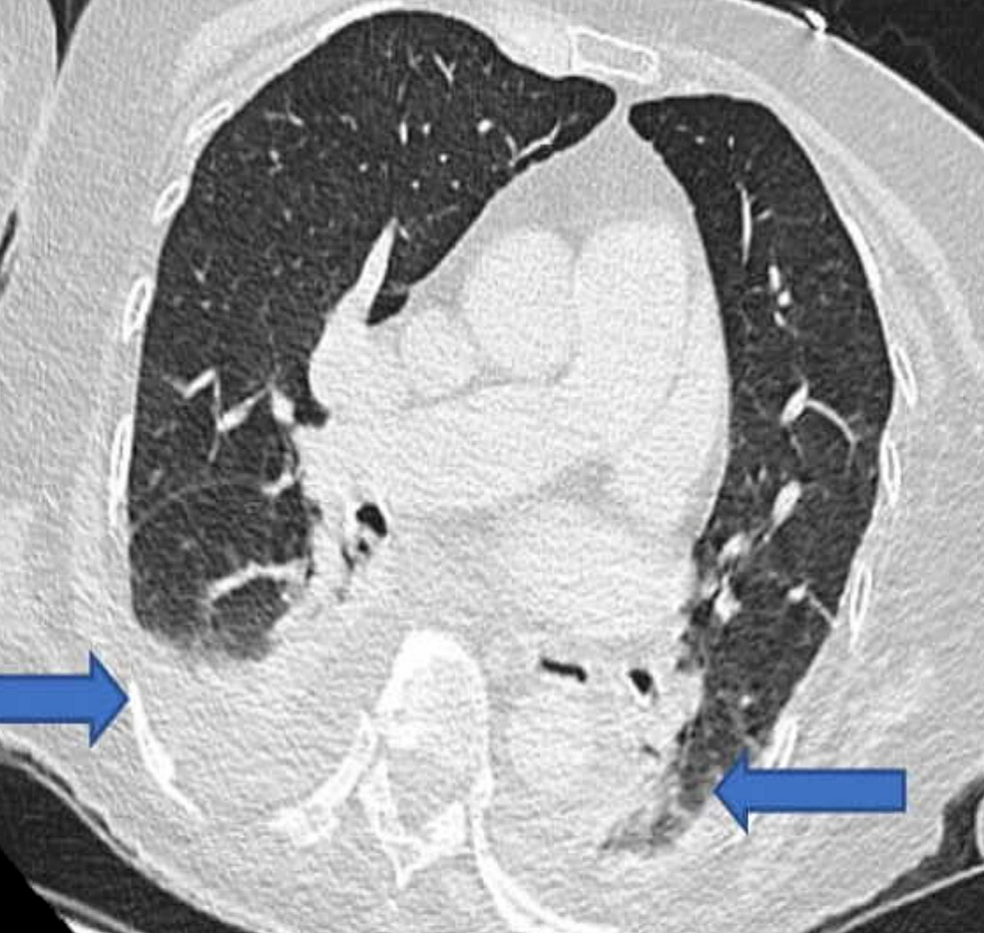
CT of the chest, abdomen and pelvis showing bilateral pleural effusions

She had CT of the chest, abdomen and pelvis (CT CAP) that showed small bilateral pleural effusions (Figure 9).

**Figure 10:**
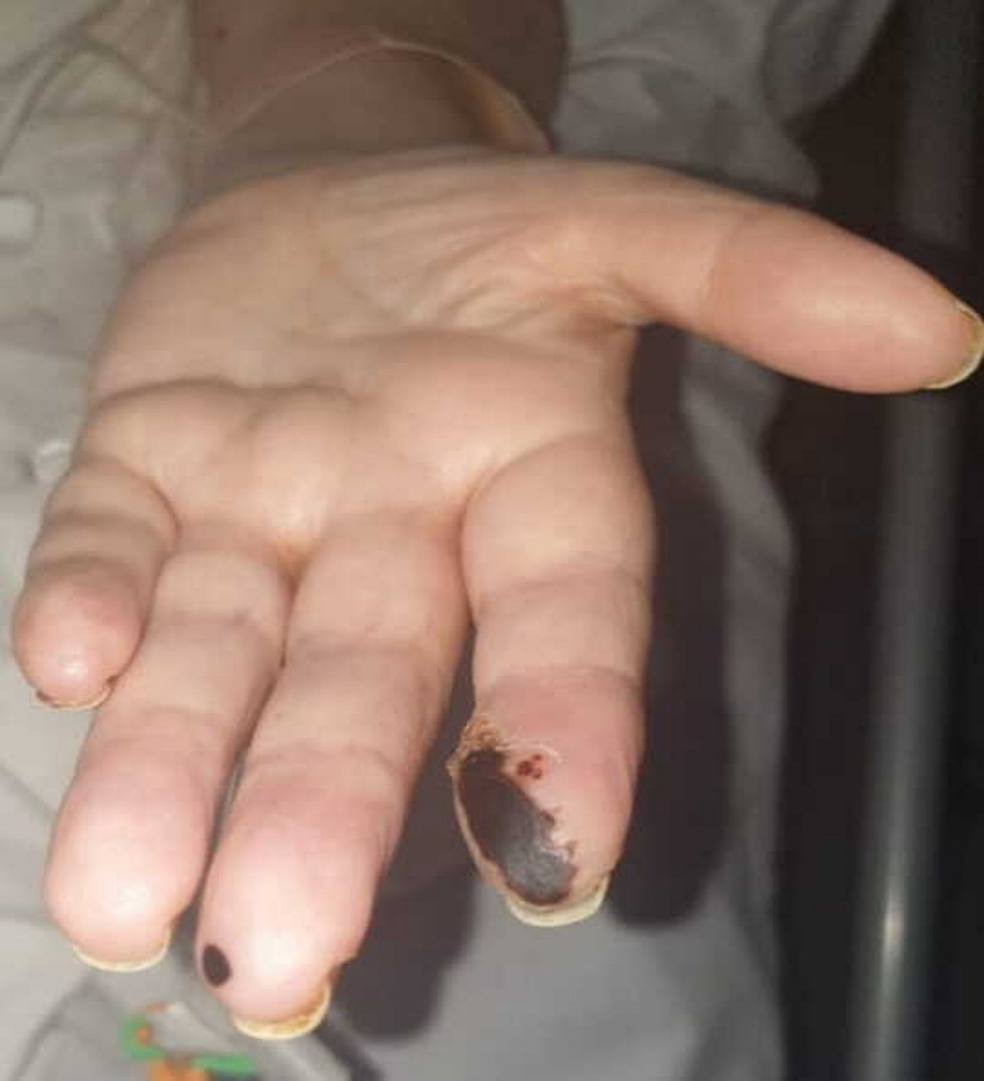
Septic emboli on patient’s digits

Subsequently, over the next few days, she developed black coloured lesions on her index and middle fingers on both hands as a manifestation of septic emboli (Figure 10).

The CT and MRI head findings were discussed with stroke and neurosurgical teams in view of her AF, and it was advised not to anticoagulate her therapeutically in view of the higher bleeding risk and to commence on prophylactic low molecular weight heparin (LMWH) dose. She was then started on prophylactic dose of LMWH in view of recurrent episodes of paroxysmal atrial fibrillation (PAF) a week after admission. This, however, had to be discontinued due to a significant drop in her platelet count below 70 × 10^9^/L. She was discussed with cardiothoracic surgeons and she was accepted for inpatient hospital transfer for tissue mitral valve replacement in view of her mitral valve endocarditis following 6 weeks course of IV antibiotics. She had successful mitral valve surgery and was referred to neurorehabilitation unit and made a good recovery. A follow-up echocardiogram after 6 months showed functioning mitral valve prosthesis.

## Discussion

Neurological complications of IE are common and well documented. Occasionally, these may be the first presenting features in patients, which include ischaemic stroke, cerebral haemorrhage, transient ischaemic attack, cerebral abscess, meningitis, and mycotic aneurysms [5]. A Finnish study reported the IE associated neurological complications to have occurred in 25% of cases [6] and 47% of these patients presented with neurological symptoms initially. About 7% of patients had cerebral haemorrhage in this study whereas other studies have reported this to 12 to 30% in patients with IE. The underlying pathophysiology of cerebral haemorrhage is not entirely clear, however, the possible explanation includes haemorrhagic transformation of cerebral ischaemic infarcts via septic emboli, rupture of pyogenic arteries and rupture of mycotic aneurysms [7]. Additionally, cerebral microbleeds have also been seen on MRI scans with the pathophysiology remaining unclear although, embolic phenomena in the vaso-vasorum or immunological vasculitis have been suggested as the likely explanation.

There are few case reports published previously on the association of IE with intracerebral haemorrhage [8,9]. One study reported that septic emboli were necessary substrate for ICH and clinically recognised ipsilateral embolism precedes ICH in only 40% of cases [10]. Additionally, they also reported that tricuspid valve endocarditis despite sustained bacteraemia, even with virulent organisms, did not result in ICH, supporting the fact that embolic fragments were necessary for it to cause ICH [9,10]. SA-associated symptomatic ICH, occurred within 48 hours of admission and other studies have reported similar findings about the association of SA-IE with early ICH [11]. In addition to SA-IE, one study reported ICH associated with *Streptococcus bovis*-IE, which according to the authors, was the first case reported worldwide [9].

Studies have shown that dilated mycotic aneurysms may be more amenable to surgical therapy, particularly when there is no evidence of acute infection and in most cases, they probably spontaneously heal without rupture with only antimicrobial treatment. This, however, does not eliminate the risk of mycotic aneurysm rupture and late rupture of the mycotic aneurysm can occur [11]. The incidence of ruptured mycotic aneurysm is about 1.7% compared to its reported prevalence in about 5% of patients with IE [10,11].

Another study based on 963 patients with left-sided IE from 2000 to 2015, reported 68 patients (7%) to have developed ICH [9]. ICH was classified into three categories based on the possible mechanism that included ruptured mycotic aneurysm, haemorrhage after ischaemic stroke and undetermined aetiology. They reported five important variables to be associated with increased risk of ICH that include platelet count < 150 × 10^9^/L, severe valve regurgitation, ischaemic stroke, symptomatic systemic embolism, and presence of a mycotic aneurysm. ICH was not associated with increased mortality in 2-3 month follow-up, although the 1- year mortality rate differed according to the ICH mechanism and it was higher for patients that did not have cardiac valve surgery when it was indicated [9,12].

This case highlights several key considerations when approaching a patient with infective and neurological symptoms, especially in smaller hospitals where it is difficult to get out of hours of echocardiogram. Therefore, it is important to be aware of the Duke criteria and consider IE as a possible differential diagnosis in patients presenting with ICH and other neurological features. Additionally, early antimicrobial initiation is key to successful treatment and improvement in mortality, possibly because this helps stabilise infective vegetations [12,13].

Non-aneurysmal spontaneous SAH as a complication of IE is infrequent and has only been described in a few case reports [14,15]. Its pathogenesis remains unknown and few suggested hypotheses include focal arteritis, vessel rupture, and spontaneous occlusion of leaking aneurysm after haemorrhage [14]. The presentation is usually non-specific making the diagnosis extremely challenging and the headache is often described by patients as diffuse and vague as opposed to the sudden severe headache classic for aneurysmal SAH [15].

**Figure 11:**
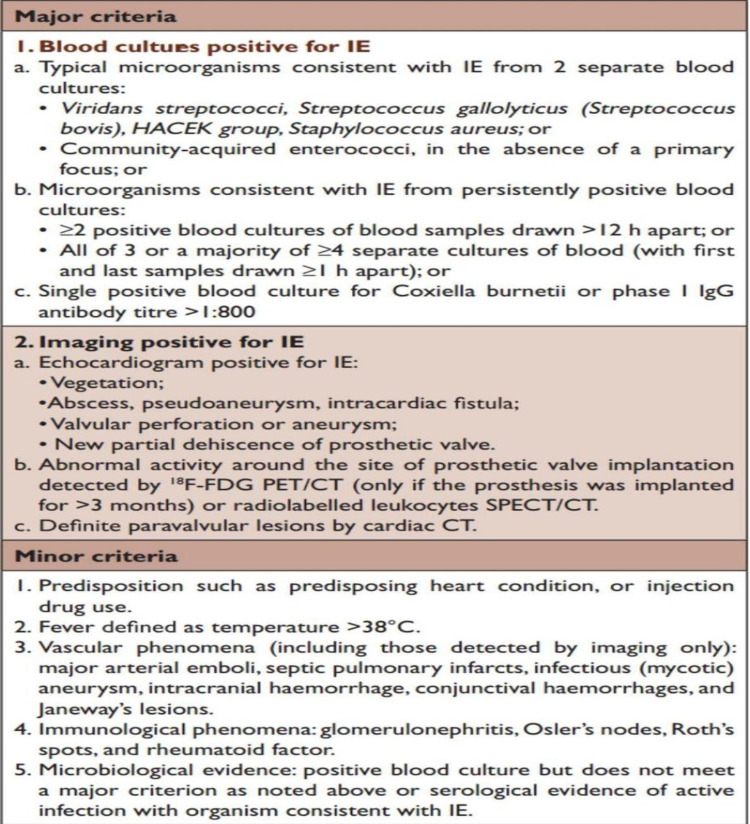
Modified Duke Criteria 2015 ESC Guidelines for the management of infective endocarditis [Internet]. Mnhums.blogspot.com. 2022 [cited 5 January 2022]. Available from: http://mnhums.blogspot.com/2015/10/2015-esc-guidelines-for-management-of.html IE: infective endocarditis; SPECT: single-photon emission computed tomography; FDG: fluorodeoxyglucose; PET: positron emission tomography; HACEK: Haemophilus species, Aggregatibacter species, Cardiobacterium hominis, Eikenella corrodens and Kingella species

**Figure 12:**
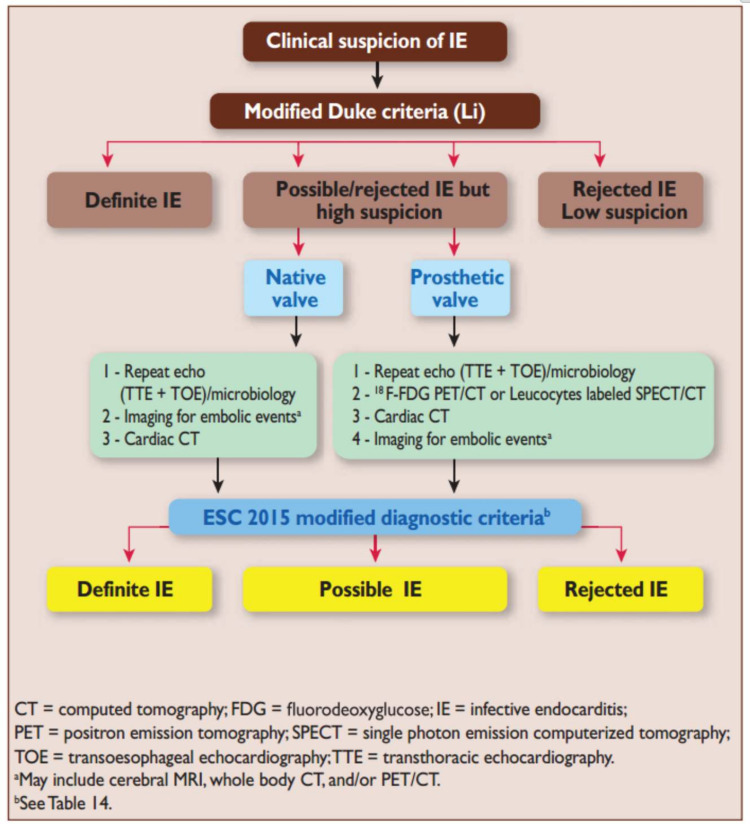
Risk stratification for IE 2015 ESC Guidelines for the management of infective endocarditis [Internet]. Mnhums.blogspot.com. 2022 [cited 5 January 2022]. Available from: http://mnhums.blogspot.com/2015/10/2015-esc-guidelines-for-management-of.html

Patients with suspected IE who are seriously unwell should ideally be managed on the intensive care unit initially in view of the higher risk to achieve a better outcome [16,17]. Another important factor in our patient was the development of AF with co-existent ICH or haemorrhagic infarcts. This can present a treatment dilemma as AF increases the risk of stroke and anticoagulation will put the patient at further risk of bleeding and it is, therefore, important to discuss these patients with stroke and neurosurgical teams. Additionally, despite no obvious evidence of an ischaemic stroke on CT or MRI, additional silent neurological events occur frequently in patients who have had neurological complications [18,19,20]. PET/CT has been shown to increase the sensitivity of the modified Duke criteria (Figures 11-12 provided in Appendices) when combined with other clinical and echocardiographic parameters and the sensitivity of contrast-enhanced CT (CECT) was 37.5% compared with PET/CT, which had a 100% sensitivity in detecting IE in the largest trial on Bentall graft infection [21]. In addition, PET/CT is a useful adjunct to the available diagnostic tools in patients with challenging cases such as patients with prosthetic valve endocarditis [22].

Studies have reported that surgery is performed in approximately 50% of cases with IE in Europe, with embolic complications being the indication in 18% of cases [20,21]. However, neurological complications complicate the timing of surgery. As such, European guidelines recommend at least 4 weeks of antibiotic therapy once cerebral haemorrhage is confirmed before performing cardiac surgery for patients with IE [23,24]. It is important to be aware of IE in patients with congenital defects such as Tetralogy of Fallot (TOF) and patent ductus arteriosus (PDA) and rare pulmonary prosthesis endocarditis that may present with right ventricular outflow tract obstruction (RVOT) and right heart failure [25].

## Conclusions

IE remains a potentially devastating condition that would likely be fatal in all untreated cases. Complicated IE should be managed in a tertiary centre specialist team. However, acute medical physicians are the often first clinicians to treat these patients and quite often need to do so in the absence of important resources, especially in district general hospitals. As such, it is important to have a low threshold of suspicion for IE in patients such as ours, as early recognition and initiation of appropriate management are essential to improve chances of survival.

Subarachnoid haemorrhage is a potentially fatal complication of IE and the presentation of headache can be completely different to the classical subarachnoid haemorrhage in these patients. It can be challenging to reach the correct diagnosis of IE in patients, particularly in absence of emergency echocardiography. This case report highlights the importance for acute medical physicians to be aware of key presenting features of IE and to consider it as one of the key differential diagnoses in unwell patients, presenting with fever and neurological symptoms.
